# State of the Art and Development Trends in Obtaining Fast-Dissolving Forms of Creatine Monohydrate

**DOI:** 10.3390/ph19010128

**Published:** 2026-01-12

**Authors:** Sabr A. Albagachiev, Elizaveta D. Pinegina, Ivan A. Sadkovskii, Ivan I. Krasnyuk, Mark A. Mandrik

**Affiliations:** 1 A.P. Nelyubin Institute of Pharmacy, Sechenov First Moscow State Medical University, 8-2 Trubetskaya Str., 119991 Moscow, Russia; albagachiev@ips.ac.ru (S.A.A.); epinegina@gmail.com (E.D.P.);; 2 A.V. Topchiev Institute of Petrochemical Synthesis, Russian Academy of Sciences, Leninsky Pr. 29, 119991 Moscow, Russia

**Keywords:** fast-dissolving creatine, modified creatine, creatine monohydrate, solubility enhancement methods, patent studies

## Abstract

Creatine monohydrate is known for its moderate solubility (13 g/L at 25 °C), which limits the feasibility of producing its fast-dissolving forms. Overcoming this limitation is possible through the application of technological approaches, the overview of which is presented in this work, including chemical modification, micronization, granulation, amorphization, formation of solid dispersions, and encapsulation. The results showed the predominance of chemical methods (about 60% of the analyzed patents). At the same time, the use of physical methods and the combination of several technologies can increase both the dissolution rate and the solubility of creatine monohydrate while maintaining its stability. This makes these approaches the most promising for the development of production technology for fast-dissolving forms.

## 1. Introduction

The sports nutrition market is demonstrating steady growth. The global sports nutrition market was valued at USD 45.24 billion in 2023 and is expected to achieve a compound annual growth rate (CAGR) of 7.5% from 2024 to 2030, reflecting the increasing demand for ergogenic aids [1]. Among these, the most sought-after supplements remain protein concentrates, BCAAs, and creatine, which optimize muscle protein synthesis and energy metabolism [2]. Studies show that up to 67% of elite athletes regularly use dietary supplements [3].

Creatine monohydrate (CM) maintains its status as the “gold standard” among ergogenic aids. Meta-analyses conducted in 2024 revealed that CM supplementation significantly increases upper and lower body strength. Specifically, for strength athletes without prior CM use, it can lead to muscle mass gains of 1.5–2 kg within 4–12 weeks and reduce body fat percentage by 0.88% [4,5]. The observed outcomes are a direct consequence of its mechanism of action, which involves enhancing ATP resynthesis through the increase in intracellular phosphocreatine stores, directly influencing muscle performance under short-term high-intensity loads [6]. Long-term studies (up to 10 years) have not identified any significant adverse effects, even with CM dosages of 20–30 g/day, confirming its safety [7]. Emerging data also highlight the therapeutic potential of creatine in neurodegenerative diseases and age-related sarcopenia, broadening its application spectrum. In rare creatine-deficient syndromes, where impaired synthesis or transport leads to severe neurological dysfunction, creatine monohydrate supplementation has been shown to partially reverse cognitive and motor deficits. Similarly, in psychiatric disorders such as depression, schizophrenia and panic disorder—conditions characterized by chronically suppressed brain creatine—supplementation restores neuronal energy homeostasis and improves clinical outcomes. After mild traumatic brain injury, creatine levels fall, and the brain enters a hypometabolic state; early open-label trials report that high-dose creatine (0.4–0.8 g/kg·day) accelerates recovery of cognition, communication, and self-care and reduces headaches, dizziness and fatigue in children. Finally, as aging impairs cerebral bioenergetic reserves, long-term creatine supplementation may help maintain cognitive function by replenishing brain creatine stores [8,9].

In parallel with its established efficacy and safety profile, the diversity of commercially available creatine monohydrate–based products has been steadily expanding. Currently, CM is predominantly marketed in powder form intended for dissolution in beverages, including instantized and micronized variants designed to improve dispersibility; however, alternative dosage forms such as tablets, capsules, effervescent formulations, portioned sachets, and, more recently, chewable and gummy matrices have also gained attention as means to improve convenience of intake and user compliance [10]. Typical recommendations in contemporary literature converge on a maintenance intake of approximately 3–5 g per day, which sets the practical dose requirement that must be accommodated by any finished dosage form. Beyond competition-driven product differentiation, the emergence of novel CM formulations is increasingly guided by technological challenges related to dose loading, dissolution behavior, and ease of administration [11]. In addition, recent studies indicate that co-administration of creatine with carbohydrates, inducing an insulin-mediated response, may enhance muscular creatine uptake and retention, further stimulating the development of combined formulations and carbohydrate-containing matrices designed to improve both usability and functional performance [12].

From a technological perspective, a key limitation of CM is its moderate solubility. The thermodynamic solubility of CM in water at 25 °C does not exceed 13 g/L, meaning that when using unmodified forms, the standard dose (5 g) does not fully dissolve in 200–300 mL of liquid [13]. As a result, even with vigorous mixing, an unstable suspension is formed, which leads to user discomfort, notably the need to continuously re-suspend the undissolved CM sediment [14]. Moreover, incomplete dissolution of CM may negatively affect its absorption rate [15].

Importantly, the intrinsic solubility of CM is a thermodynamically defined property and cannot be substantially increased without chemical modification of the molecule, which formally results in new chemical entities rather than creatine monohydrate itself.

Therefore, fast-dissolving creatine formulations should be viewed primarily as technological solutions aimed at overcoming practical solubility limitations by accelerating dissolution kinetics and enabling the transient formation of supersaturated or metastable solutions, rather than altering equilibrium solubility.

In this context, the development of fast-dissolving forms of creatine becomes increasingly relevant. Reducing dissolution time not only promotes faster entry of the active substance into the bloodstream and uptake by muscle cells but also expands opportunities for creating new sports nutrition products, which are in growing demand [13].

The aim of this study is to conduct a patent and bibliographic analysis of technological approaches for overcoming solubility-related limitations in the development of fast-dissolving forms of creatine monohydrate.

## 2. Technologies for Overcoming Solubility-Related Limitations of Creatine

### 2.1. Chemical Modification

In the context of improving the dissolution behavior of creatine monohydrate (CM), chemical modification occupies a specific position. Unlike physical modification methods applied directly to CM, chemical modification involves structural changes to the creatine molecule, which formally result in creatine derivatives rather than CM itself. In this review, these derivatives are not considered independent active substances. Instead, they are discussed as technological strategies that address the intrinsic solubility limitations of CM by altering its molecular structure, formally yielding creatine derivatives with modified physicochemical properties and improved formulation performance.

Chemical modification involves targeted alteration of the molecular structure of a substance by introducing functional groups, substituents or through complexation and salt formation (i.e., forming new chemical bonds). Such modifications allow for adjusting the physicochemical and biopharmaceutical properties of compounds, including solubility, stability, and bioavailability [16]. In the fields of pharmaceuticals and nutraceuticals, improving solubility is particularly important, as it directly affects the rate and extent of active ingredient absorption in the body [17].

Chemical modification enables changes in the physicochemical properties of a substance, thereby facilitating its rapid dissolution [18]. The versatility of this method lies in the ability to select functional groups to tailor the compound’s properties for specific conditions such as pH and temperature [19].

Regarding the creatine molecule, various chemical modification strategies have been proposed to alter its physicochemical and formulation-related properties. The patent literature describes creatine salts such as hydrochloride, α-ketoglutarate, β-alaninate, carbonate, bicarbonate, pyroglutamate, and glycinate, all exhibiting improved solubility compared to the native form [20,21,22,23,24,25,26,27]. Attention has also been paid to salts of creatine derivatives. For example, creatine ethyl ester forms compounds with organic acids (malic, fumaric, pyruvic), as well as salts with iminosugars presented as hydrochlorides, hydrobromides, sulfates, hydrosulfates, trifluoroacetates, citrates, hydrocitrates, dihydrocitrates, maleates, hydromaleates, malates, hydromalates, fumarates, and hydrofumarates [28,29]. Additionally, double salts of creatine have been developed, where cations include sodium, potassium, ammonium, L-lysine, L-arginine, or ethanolamine, and anions include sulfate, phosphate, L-aspartate, L-glutamate, malonate, succinate, fumarate, or glutarate [30].

Esterification of creatine and its derivatives is also used to improve their properties. Examples include salicylate esters of creatine phosphate, and the creation of prodrugs based on esters synthesized by reacting creatine with alcohols (e.g., ethanol, benzyl alcohol, glycerol) [31,32].

Additional modifications include reactions of creatine and its derivatives with esters or amides of glycine and lysine, iminosugars, acyl halides and other acylating agents, leading to N-acetylcreatine, N,N-diacetylcreatine, creatyl peptides and other acetylated derivatives [33,34,35,36]. Among phosphorylated forms, creatinol-O-phosphate, phosphocreatine disodium, as well as hybrid structures such as creatine ascorbyl phosphate and phenyl creatine are noteworthy [37,38,39,40].

To improve organoleptic properties, particularly to mask bitterness, creatine salts with saccharinate or acesulfamate have been proposed [41]. Some patents also describe compounds not directly focused on solubility enhancement, such as creatine salts with hydroxycitric acid, heterocyclic acids (orotic, thioorotic, dihydroorotic), and dicarboxylic acids (maleic, malic, fumaric, tartaric) [42,43,44].

Despite the broad range of compounds described in patent literature, this list is far from exhaustive. The scientific literature mentions other forms of creatine, including anhydrous creatine, magnesium creatinate, methylamino creatine, creatine methyl ester hydrochloride, creatine nitrate, tricreatine citrate, dicreatine citrate, creatine citrate, creatine pyruvate, creatine lactate, creatine sulfate, ethyl ester pyruvate creatine, methyl ester hydrochloride creatine, sodium creatine phosphate, creatine malate, creatine glutamate, ethyl ester malate creatine, 5-hydroxytryptamine creatine, creatine trinitrate, D-gluconate creatine, creatine phosphate lactate, creatine-CoA, creatyl-L-leucine, and guanidinoacetic acid as a precursor [11,13].

Enhancing lipophilicity represents another strategy to improve the bioavailability of creatine and its delivery to the brain. For example, Chan et al. propose the synthesis of lipophilic prodrugs of creatine. Modified creatine forms are synthesized by introducing amide groups with alkyl (C_6_–C_20_) or alkenyl (C_6_–C_20_) chains, increasing lipophilicity and the ability to overcome biological barriers via passive diffusion. Intramolecular cyclisation is also performed via olefin metathesis, followed by the synthesis of stable salts (e.g., hydrochloride, trifluoroacetate), improving structural stability and physicochemical properties [45].

Despite the wide range of chemical modifications, data on the actual solubility enhancement and clinical effectiveness of these new creatine forms are limited. For most derivatives, independent in vivo studies evaluating their bioavailability and potential side effects (e.g., gastrointestinal irritation or acid-base balance changes) are lacking. Therefore, further clinical trials are needed to confirm their advantages and assess the associated risks.

Reported aqueous solubility values of creatine salts, creatine derivatives, and salts of creatine derivatives are summarized in Table 1.

### 2.2. Physical Modification and Formulation

#### 2.2.1. Micronization

Micronization of CM is a process of reducing particle size to the micron (1–10 μm) or nanometer (<100 nm) range, which significantly enhances the dissolution rate and apparent solubility due to the increased surface area. To achieve this, both mechanical dispersion and physicochemical methods are employed. The former includes jet and ball mills as well as high-pressure homogenization. A drawback of these methods is that they can induce particle aggregation due to the accumulation of electrostatic charge. In contrast, approaches such as spray drying, precipitation from supercritical fluids (SCF), and in situ micronization using hydroxypropyl methylcellulose (HPMC) enable the production of particles with controlled morphology [50].

Several technological approaches to micronizing CM have been proposed in patents, aimed at enhancing dissolution behavior, apparent solubility, stability, and bioavailability.

Japanese researchers led by Katsukita described wet milling in a bead mill using a non-aqueous solvent (e.g., ethanol) and beads up to 1 mm in diameter, producing particles ≤2 μm. The process involves dispersing creatine in the solvent, milling under cooling to prevent thermal degradation, subsequent bead separation, and drying. As a result, the apparent solubility of creatine at 10 °C reaches 2.11%, more than twice that of non-micronized forms; additionally, stability in acidic environments and reduced conversion to creatinine during storage were confirmed, along with accelerated absorption during transdermal and sublingual administration [51].

Stitley and Anthony patented dry milling of CM in an impact mill with the addition of a water-soluble agent, such as dextrose, in a 1:2 to 1:6 ratio. According to the patent, the resulting powder with particle size up to 40 μm (preferably up to 10 μm) dissolves in cold water (12–15 °C) in no more than 5 min; with shaking, the process can take around 10 s, forming a vissually clear solution containing 5 g of creatine per 532.26 mL (18 oz) of water. The use of the water-soluble agent prevents agglomeration, while temperature control (not exceeding 28 °C) minimizes degradation; the technology also allows the introduction of flavorings and colorants without loss of stability [52].

The following technology is a combined approach focusing on optimizing particle size in the range of 5–200 μm to achieve a balance between texture and prevention of sticking, using non-reducing sugars (sucrose, erythritol). Here, the emphasis is not on modifying intrinsic solubility but on eliminating grittiness, preventing the Maillard reaction, and ensuring stability under extreme storage conditions (50 °C, 90% humidity). The process includes micronization followed by granulation at temperatures not exceeding 80 °C, drying to a moisture content of 7.5–16%, and pressing at pressures from 100 to 8000 kg/cm^2^, which preserves the consumer qualities of creatine and promotes rapid glycogen replenishment upon use [53].

For CM micronization, the RESS (Rapid Expansion of Supercritical Solutions) method was also employed: the substance was first dissolved in supercritical CO_2_, then pressure was rapidly reduced by passing the solution through a nozzle. This caused a rapid expansion of the solution, instant nucleation, and precipitation of fine creatine particles. During the experiment, the extraction temperature (39–60 °C), pressure (14–22 MPa), nozzle length (2–15 mm), effective diameter (450–1700 μm), spray distance (1–7 cm), and other parameters were varied. As a result, the mean particle size decreased from 44.67 μm to a range of 0.36–9.06 μm. Shortening the nozzle length and diameter and increasing the extraction pressure favored the formation of smaller particles, while increasing the spray distance broadened the size distribution. Scanning electron microscopy revealed that the processed particles acquired a spherical shape instead of the original needle-like structures, and X-ray diffraction analysis showed reduced crystallinity. The appearance of a polymorphic form of CM was observed during the experiment, but adjusting the pressure helped eliminate this phenomenon [54].

Despite the advantages of micronization—enhanced dissolution rate and increased apparent solubility—the resulting powders remain physically unstable. Recrystallization, changes in physicochemical properties during storage, and a tendency toward agglomeration limit their use in finished products [45]. One solution to this problem is combining micronization with subsequent granulation, which not only enhances the flowability and processability of powders but also stabilizes the particle structure, preventing degradation of the active ingredient, as demonstrated in one of the patents [52]. This approach enables the development of creatine forms that combine rapid dissolution and high apparent solubility of micronized particles with long-term stability and user convenience.

#### 2.2.2. Granulation

Granulation is a process of particle enlargement by agglomeration and is one of the key operations in the production of solid forms. During granulation, fine powders are transformed into free-flowing, dust-free granules with properties that differ significantly from those of the original powder. There are two main types of granulation: wet granulation, which involves the use of a binder liquid, and dry granulation, which does not require liquid; the choice of method is determined by the analysis of the physicochemical properties of the active ingredient, excipients, and the requirements for the final product [55].

This technology addresses practical limitations stemming from poor dissolution behavior, physical instability, and formulation-related variability in absorption at creatine loading doses [14].

For instance, a patent owned by the German company ‘AlzChem Trostberg GmbH’ (Trostberg, Germany) describes a method for creating readily dispersible agglomerates in which ground creatine or its salts (30–99.9%) are combined with maltodextrin (0.1–30%) as a binding agent. Using granulation, extrusion, or fluid bed processing, particles with improved dissolution rates, reduced dust formation, and enhanced flowability are obtained, addressing the poor dissolution behavior of crystalline creatine and the drawbacks of micronized forms, such as sediment formation upon dissolution [56].

Byrd patented a process for producing creatine ester granules containing dicalcium phosphate, polyvinylpyrrolidone, starch, and magnesium stearate, designed for tableting. This formulation ensures prolonged release over 4–8 h and maintenance of blood creatine concentrations at 50–300 μg/mL, while particle coatings mask taste and extend shelf life. However, the authors did not report improvements in solubility [57].

Similarly, in 2025, Tempesta and Daugherty patented combining disodium phosphocreatine with cyclodextrins (α-, β-, γ-types) and excipients (PEG 3350, starch, trisodium citrate) to protect CM from degradation in the stomach and ensure intestine-specific release. Additionally, the granules are coated with a methacrylate-based layer (Eudraguard^®^ (Evonik, Essen, Germany)) with a thickness of 150–850 μm, ensuring acid resistance and a 1.5-fold increase in in vitro permeability parameters, although no enhancement of solubility was reported [58].

Another approach involves granulating CM (2.5 g/kg) with leucine (35 mg/kg) in a sugar matrix with crystal sizes of 0.20–0.30 mm. The process includes granulation using a sugar solution (82–83% solids at 80–90 °C) and drying at 110–115 °C, preventing clumping and ensuring uniform additive distribution. However, this method focuses on thermal stability and structural uniformity rather than modification of intrinsic solubility [59].

Thus, the data suggests that only one of the four reviewed patented granulation methods directly targets overcoming solubility-related limitations of creatine by enhancing dissolution behavior, while the other approaches focus on controlling release, ensuring stability, and uniform mixing of components.

From a mechanistic perspective, the positive effect of granulation on dissolution behavior can be attributed to several interrelated factors, including improved powder wettability, reduced interparticle cohesion, and enhanced liquid penetration into the solid matrix. Granulation also leads to a more uniform particle size distribution and increased porosity, which facilitates faster disintegration and dissolution [60,61]. In addition, the use of binders and hydrophilic excipients during granulation may contribute to transient supersaturation effects and delay recrystallization, thereby improving apparent solubility and dissolution kinetics without altering thermodynamic solubility [62,63].

#### 2.2.3. Amorphization

Amorphization is the process of converting a crystalline material into the amorphous state, characterized by the disordered arrangement of molecules [64]. This results in weaker intermolecular interactions and an increase in Gibbs free energy in the amorphous form in comparison with the crystalline state, which enhances the dissolution rate of the substance and facilitates the formation of transient supersaturated states in aqueous media [65]. These supersaturated solutions represent a metastable, kinetically driven state rather than an increase in thermodynamic solubility.

Despite the advantages of amorphization, its application is limited by two factors: the inherent thermodynamic instability of amorphous forms and the risk of recrystallization. This can occur both in the solid state during storage and in solution after reconstitution. Supersaturated solutions are prone to solution-mediated recrystallization into the thermodynamically stable crystalline phase, which highlights the need for combining amorphization with other technological methods addressed to suppress nucleation and crystal growth. A prevalent strategy involves incorporating the drug into amorphous solid dispersions (ASDs) using polymer matrices. This offers partial solution to these limitations; however, their hygroscopicity and often limited solubility drive the development of other combined approaches [66]. Notably, existing patent solutions in the field of amorphization are scarce, likely due to the technical challenges associated with long-term stabilization of the amorphous state and the greater practical potential of alternative technological methods.

In one patent, the key issues of poor dissolution behavior and physical instability of CM in aqueous media are addressed by producing a fast-dissolving powder combining creatine monohydrate (18–40%) with solubility enhancers (sucrose, glucose), stabilizers (microcrystalline cellulose, xanthan gum), and carriers (maltodextrin). The process involves high-speed dispersion of the component mixture with water (10,000–22,000 rpm), followed by homogenization (30–100 MPa) or microfluidization (30–80 MPa) of the resulting suspension, and subsequent spray drying and granulation. This approach reduces particle size and stabilizes them in an amorphous/microcrystalline form, leading to a 3–10-fold increase in apparent (kinetic) solubility, while preserving biological activity and suspension stability for up to 3 days [67].

Runchevski et al. proposed a mechanochemical synthesis of a co-amorphous form of creatine with citric acid (a-CCA), achieved by dry milling of equimolar quantities of the component anhydrides, with its subsequent transformation into a more stable co-crystalline form [68].

Neiss describes a technology for creating a solid dispersion of creatine in a protein matrix, produced by spray drying an aqueous solution containing CM, a protein component (whey concentrates, isolates), and minerals (sodium, potassium, calcium, and magnesium salts). The resulting powder, with particle sizes of 10–250 μm, features uniform distribution of amorphous creatine in the matrix, improving dissolution behavior, apparent solubility, and physical stability [69].

Research results indicate that, although amorphization can be used to enhance the dissolution rate and apparent solubility of creatine through supersaturation mechanisms, using this approach alone generally does not provide all the required product characteristics. Additional technological steps such as granulation or the formation of solid dispersions are necessary to ensure stability and functionality. This highlights the necessity for an integrated, formulation-driven approach in this case [66,69].

#### 2.2.4. Solid Dispersions

The term “solid dispersion” refers to a group of solid products consisting of at least two different components, typically a matrix and an active substance. The matrix may be crystalline or amorphous, while the active substance within the matrix may be dispersed at the molecular level [70].

Several technological approaches have employed this principle to enhance the dissolution behavior, apparent solubility, and stability of creatine.

A team led by Daugherty proposed a composition containing creatine hydrochloride and polyethylene glycol (PEG) with an enteric coating. In this technology, selecting the molecular weight of PEG and optimizing its ratio to creatine are aimed at minimizing the degradation of the active substance in the gastrointestinal tract, thereby reducing creatinine formation. The use of a controlled-release mechanism is intended to support effective creatine absorption at a reduced dosage [71].

Kessel et al. proposed a method based on melt plasticization. Creatine is mixed with a thermoplastic polymer and water, followed by heating above the softening temperature of the polymer. Partial evaporation of moisture during heating leads to the formation of a stable matrix system in which creatine is uniformly distributed within the polymer matrix. This technology enables controlled release rates of the active substance and ensures its stability in solid forms [72].

A non-classical dispersion was described by Petersen et al. In this method, creatine is milled to particle sizes ≤ 160 μm and mixed with protein components, resulting in uniform distribution of the active substance within the matrix. Suspending this mixture in an aqueous medium followed by drying fixes the structure, preventing aggregation and creatinine formation during processing and storage. The use of a protein component instead of a traditional polymer makes this approach atypical for classical solid dispersions [73].

#### 2.2.5. Encapsulation

In this context, the term “encapsulation” does not refer to a classical dosage form but to micro-sized particles designed to encapsulate active substances within protective matrices. Such delivery systems can enhance dissolution behavior, apparent solubility, and physical stability of substances, and enable controlled or targeted release. The size of these systems varies: nanocapsules typically have diameters ranging from 10 to 1000 nm, while microcapsules range from 0.2 to 5000 μm, allowing their application in various medical and food technology domains. Structurally, capsules consist of a core containing the active ingredient (e.g., a drug or nutrient) surrounded by polymeric, lipid, or other types of shells [74,75,76].

Various materials are used to create capsules, including synthetic polymers (e.g., PLGA), natural biopolymers (chitosan, starch), lipids, and many others. Methods of capsule production include interfacial polymerization, emulsion–diffusion techniques, and sol–gel technologies, which determine the properties of the capsules, including size, release kinetics, and stability [74,75,76,77,78].

Krolevets patented a method for producing creatine nanocapsules with a gellan gum shell. The process involves dispersing creatine in a suspension of the polymer in hexane, using a surfactant (mixed esters of citric and edible fatty acids with glycerol, known as food additive E472c), followed by shell deposition with ethyl acetate. The core-to-shell ratio can be varied (1:1 or 1:3), and the synthesis time is reduced to 20 min by eliminating steps requiring UV irradiation or specialized equipment. The yield of nanocapsules reaches 100% by weight, and the use of non-toxic components (gellan gum, ethyl acetate) minimizes risks associated with synthetic polymers [79].

In another patent, also by Krolevets et al., a technology for encapsulating creatine in microcapsules with a sodium alginate shell is described. The method relies on physicochemical precipitation by a non-solvent: a suspension of creatine monohydrate in benzene is mixed with butanol containing sodium alginate and surfactant (mixed esters of citric and edible fatty acids with glycerol), followed by chloroform addition to precipitate the shell, and drying. The process is conducted at room temperature without sophisticated equipment, ensuring, according to the authors, 100% yield of microcapsules within 20 min [80].

Beyond these patented approaches, other methods for encapsulation of creatine derivatives have been reported in the scientific literature. One of them involves preparation of lipid nanocapsules with dodecyl creatine ester. Creatine dodecyl ester was previously dissolved in Transcutol^®^ (Gattefossé SAS, Saint-Priest, France) at room temperature, which prevented its hydrolysis at higher temperatures and ensured uniform distribution in the subsequent lipid shell of the nanocapsules. Due to the introduction of the dodecyl group, the molecule becomes significantly more lipophilic, which facilitates its penetration through biological barriers, particularly the blood–brain barrier (BBB), improving creatine local brain bioavailability. It also makes dodecyl creatine ester a promising candidate for drug development as a therapeutic option for patients suffering from creatine transporter deficiency. Nanocapsules, when administered parenterally, additionally protect ether from rapid destruction by plasma esterases and direct it to the brain endothelium. In vitro studies on fibroblast cell lines with creatine deficiency demonstrated that dodecyl creatine ester is released from the nanocapsules and undergoes intracellular biotransformation to active creatine. Other in vitro studies using a blood–brain barrier model showed that lipid nanocapsules penetrate brain endothelial cells via transcytosis. The main aim of the authors of this method was not enhancing creatine solubility but improvement of BBB penetration for potential neurodegenerative disease therapies [81].

Another method reported in scientific studies involves encapsulating CM in polymeric nanoparticles using the double emulsion (W/O/W) technique, designed to address formulation challenges associated with limited dissolution behavior in aqueous and organic media. This method is a two-step process: first, an aqueous creatine solution is emulsified in an organic phase (W/O), forming particles with a water core; then, the primary emulsion is dispersed in an external aqueous phase (W/O/W), creating nanoparticles with a double structure—a hydrophilic core containing creatine and a polymeric shell. This method effectively encapsulates creatine, improving its dissolution behavior and protecting it from premature gastrointestinal exposure, as well as enabling modulation of release profiles through polymer composition [82].

#### 2.2.6. Other Technological Approaches

The previous sections have summarized the main technological approaches to addressing solubility-related limitations of creatine. However, the scientific and patent literature also describes other, less common methods that cannot be separated into distinct sections due to limited research volume yet deserve attention because of their specificity. Some of these approaches are based on combinations of known processes, while others propose original but little-studied solutions. The analysis of such approaches reveals additional opportunities for optimizing creatine properties—ranging from reducing dissolution time and improving physical stability to modulating release behavior and, in some cases, influencing bioavailability. This chapter is dedicated to these technologies.

The previously mentioned patent, concerning the mechanochemical synthesis of the co-amorphous form of creatine with citric acid (a-CCA), also describes a method for obtaining its co-crystalline form (c-CCA). The study demonstrates how changing processing conditions (moisture introduction) allows switching between amorphous and crystalline phases. To form the c-CCA co-crystal, moisture is added to the mechanochemical process either through the use of hydrated forms of the starting components or by dropwise addition of water. This results in a monoclinic structure (space group P2_1_/c), where the asymmetric unit contains creatine and citric acid molecules in a 1:1 ratio. This transition from the amorphous to the crystalline state is confirmed by X-ray diffraction with Rietveld refinement, IR spectroscopy, thermogravimetric analysis (TGA), and DFT calculations. Interestingly, the co-amorphous a-CCA form, which is stable in dry conditions, transforms into the ordered c-CCA co-crystal upon moisture exposure. The c-CCA co-crystal, representing a distinct multicomponent crystalline phase, exhibits improved water solubility (42.1 g/L), three times higher than that of CM, and retains thermal stability up to its melting point with decomposition. Controlling humidity during synthesis opens the way to creating materials with tailored properties, which is particularly relevant for developing formulations with tailored physicochemical properties and, potentially, enhanced bioavailability. This patent not only demonstrates the dual nature of the mechanochemical approach but also underscores the role of external factors in controlling phase transitions [68].

A lyophilized preparation based on sodium phosphocreatine has also been developed, in which physical stability and dissolution rate are improved through optimized composition and production technology. The addition of excipients such as dextranes, sugars, polyols or sodium chloride in specific mass ratios (1:0.05–1:20) stabilizes the active ingredient during lyophilization, which includes multi-stage freezing and drying under controlled temperature conditions. Treatment with activated carbon and sterile filtration minimizes its pyrogen content, while a closed process reduces the risk of contamination. The resulting product demonstrates reduction in dissolution time to 30–60 s and maintains key parameters (active substance content, impurity levels) during long-term storage under various conditions, as confirmed by 24-month tests [83].

Another approach involves solubilization strategies for creatine derivatives intended for parenteral administration. Thus, work of Wang Le describes a combination of creatine phosphate with arginine as a solubilizer in a specific ratio (1:0.8–1:2.5), which provides an acceptable pH in solution and reduces irritation of blood vessels, compared with the commonly used sodium salt of creatine phosphate. The absence of sodium in the composition broadens its application for patients with sodium intake restrictions, while the physical mixing of components under sterile conditions guarantees uniformity and stability. Experimental data demonstrate that this composition outperforms traditional forms in thermal stability and retains activity in infusion solutions for up to 8 h with minimal degradation [84].

## 3. Dissolution Kinetics as a Criterion for Fast-Dissolving Creatine Forms

Despite the substantial number of published studies and patented solutions devoted to the development of various creatine forms marketed as fast-dissolving, quantitative data directly characterizing their dissolution kinetics remain extremely limited in literature. In most cases, claims of improved solubility are based on thermodynamic solubility values, qualitative observations, or indirect indicators (e.g., visual clarity of the solution), without a systematic evaluation of the dissolution rate as an independent parameter.

Furthermore, a considerable proportion of studies in this field are primarily focused on pharmacokinetic aspects [85,86].

While such data are undoubtedly of practical relevance, they are generally not accompanied by a detailed analysis of the intrinsic dissolution kinetics of the solid form. This lack of kinetic characterization complicates the establishment of a direct relationship between the physicochemical properties of the formulation and the observed pharmacokinetic effects.

A further significant limitation in the analysis of the available data is the methodological heterogeneity of the experimental approaches employed. Across different studies, non-comparable dissolution conditions are used, including variations in temperature, medium volume and composition, agitation intensity, as well as the physical form of the sample. As a result, direct comparison of dissolution-related outcomes between studies becomes methodologically inappropriate, even when identical chemical forms of creatine are investigated.

In this context, it is essential to emphasize that an objective evaluation of fast-dissolving creatine forms requires the combined consideration of dissolution kinetics and apparent solubility. Dissolution kinetics describes the rate at which the substance transfers into the dissolved state, whereas apparent solubility reflects the concentration achieved in the dissolved phase within a relevant time interval. The joint analysis of these parameters makes it possible to distinguish formulations that merely exhibit a high equilibrium solubility from those that enable the rapid attainment of high dissolved creatine concentrations over a short period of time.

Only a limited number of publications in the available literature provide quantitative data that enables direct analysis of creatine dissolution kinetics. Notably, in the study by Ganguly et al. and in the patent US11166979B1, the dissolution of different creatine forms was investigated under controlled conditions with time-resolved monitoring of the amount of dissolved substance [87,88]. The resulting linear relationships between time and the amount of dissolved creatine enabled the calculation of dissolution rate constants, which are summarized in Table 2 of the present work.

It should be emphasized that these studies do not cover the full diversity of creatine forms reported in the scientific and patent literature. Nevertheless, the quantitative kinetic data obtained therein represent rare and reproducible examples of such measurements and may therefore serve as valuable reference points for the further investigation of fast-dissolving creatine formulations.

## 4. Materials and Methods

The patent analysis was conducted based on data from open sources, including national and international registries: Rospatent, United States Patent and Trademark Office (USA), Espacenet (European Patent Office), CIPO’s Canadian Patent Database (Canada), Lens.org, and Google Patents. To ensure comprehensive coverage, patents in Russian, English, Chinese, and Japanese were considered; however, Chinese and Japanese texts were included only if an English abstract was available. European patents in German, French, Spanish, and Italian were analyzed in their translated English versions available in the databases.

Relevant patents were identified using a combination of keywords: creatine in conjunction with solubility, rapid-dissolving, enhancement, and instant, reflected in the search query: creatine AND (solubility OR rapid-dissolving OR enhancement OR instant). To refine the results, IPC/CPC codes A23L (food additives) and A61K (pharmaceutical forms) were applied. The search was conducted in three stages: without filters, with sequential use of the codes, and with their combination. The analysis period covered applications from 2000 to 2025, including granted patents and pending applications, to capture experimental technologies.

The selection criteria focused on patents where CM was the primary component. Duplicates were eliminated by selecting the earliest version of a patent when the authors and content coincided, as well as by replacing patent families with original applications. The geographic scope included a full analysis of patents from the USA, Europe, Russia, and Canada, while Japanese and Chinese documents were considered selectively—only those with English abstracts. As a result of multi-stage filtering, the initially retrieved thousands of results were narrowed down to 43 patents that met the criteria.

The obtained data were classified according to the solubility enhancement technologies described in the patents: micronization, granulation, encapsulation, amorphization, etc. Many technologies involved combinations of these methods. To identify key trends, citation analysis was used, allowing the determination of the most influential patents. Cross-verification between databases and testing of synonyms (quick-dissolving instead of rapid-dissolving) confirmed the completeness of the selection, although language restrictions may have excluded some Chinese and Japanese patents. It should be noted that a significant portion of European, Canadian, and Russian applications turned out to be duplicates of US patents, underscoring the leadership of the USA in this field.

Following the patent analysis, a review of scientific literature was conducted using the RSCI, PubMed, Scopus, and Google Scholar databases with the same keyword queries as in the patent search.

Subsequently, an additional bibliographic and patent search was conducted directly on technological methods (creatine + chemical modification/micronization/granulation/amorphization/solid dispersions/microencapsulation/nanoencapsulation/crystallization/lyophilization/spray drying/fluid bed drying).

The methodology combines quantitative and qualitative approaches; however, the limitations are connected with the dependence on the availability of translations and the high degree of duplication in international registries.

To systematize the data obtained during the patent analysis, a summary table was compiled, covering the key patent developments over the past 25 years. The table is structured by technological methods, patent numbers, and key characteristics (Appendix A, Table A1).

## 5. Conclusions

Emerging data underscore the therapeutic potential of creatine monohydrate beyond its role as a sports supplement, while experiments indicate its efficacy in neurodegenerative diseases and age-related sarcopenia. In rare creatine deficiency syndromes, where impaired synthesis or transport results in severe neurological dysfunction, creatine supplementation has been shown to partially reverse cognitive and motor deficits. At conditions characterized by chronically suppressed brain creatine levels, such as depression, schizophrenia, and panic disorder, supplementation restores neuronal energy homeostasis and improves clinical outcomes. Following mild traumatic brain injury, a state associated with declining creatine levels and cerebral hypometabolism, early open-label trials report that high-dose creatine administration accelerates recovery of cognition, communication, and self-care abilities and reduces headaches, dizziness, and fatigue in pediatric populations. Furthermore, as aging impairs cerebral bioenergetic reserves, long-term creatine supplementation may contribute to maintaining cognitive function by replenishing brain creatine stores. This expanding therapeutic relevance underlines the critical need to address the practical limitations associated with the dissolution behavior of CM to realize its full clinical potential.

The conducted study has allowed the systematization of technological approaches aimed at addressing solubility-related limitations and enhancing the dissolution behavior of CM—key challenges in developing more effective sports supplements and pharmaceutical products based on creatine. Analysis of patent and scientific data has revealed the dominance of chemical modification (around 60% of analyzed patents and applications) as a method capable of increasing equilibrium solubility through alteration of the molecular structure of the starting material. However, despite their effectiveness, such methods can affect the stability and bioavailability of the substance and require additional safety studies. Physical methods demonstrate potential in enhancing dissolution rate and apparent solubility without altering the chemical composition but face challenges related to recrystallization, agglomeration, limited technological reproducibility, and the high cost of equipment.

Alternative solutions, including nanocapsulation, co-crystallization with citric acid, and lyophilization, offer new opportunities to overcome barriers and optimize release behavior, although their industrial implementation requires further research.

The most promising approaches are combined strategies, such as amorphization followed by granulation or preparation of solid dispersions in polymeric matrices. These methods combine the benefits of physical processing (increased surface area) and particle stabilization, ensuring rapid dissolution, transient supersaturation, and long-term physical stability.

The results presented in this work highlight the growing interest in fast-dissolving forms of CM. Optimizing dissolution behavior and formulation-driven performance of creatine will enable the development of stable products with modified release profiles and more convenient administration forms.

## Figures and Tables

**Table 1:** Reported aqueous solubility of creatine salts, creatine derivatives, and salts of creatine derivatives. The table includes only explicitly reported solubility values from scientific publications and patent documents. All data are expressed in g/mL for comparability.

Creatine Form	Aqueous Solubility (g/mL)
Creatine monohydrate	0.0133 [13]
Creatine hydrochloride	0.679 ± 0.018 [20] 0.709 ± 0.007 [46]
Creatine β-alaninate	0.0266 to 0.3325 [22]
Creatine carbonate	0.03 [24]
Creatine bicarbonate	0.0266 to 0.3325 [25]
Creatine pyroglutamate	0.0267 [26]
Creatine glycinate	0.0286 [27]
Sodium creatine sulfate	0.080 [30]
Potassium creatine sulfate	0.080 [30]
Creatine maleate	0.0374 ± 0.0073 [46]; 0.19 [47]
Creatine fumarate	0.03 [47]
Creatine tartarate	0.085 [47]
Creatine malate	0.045 [47]
Tricreatine citrate	0.029 [11]
Creatine dihydrocitrate	0.1 [47]
Creatine pyruvate	0.054 [11] 0.0916 ± 0.0077 [46]
Dicreatine sulfate	1.370 [11]
Creatine hemisulfate	0.121 ± 0.001 [46]
Creatine mesylate	0.588 ± 0.008 [46]
Dicreatine maleate	0.1596 [48]
N-acetylcreatin	0.400 [34]
Creatinol-O-phosphate	0.005 [11]
Creatine ethyl ester hydrochloride	0.2059 ± 0.0015 * [32]
Creatine benzyl ester hydrochloride	0.08926 ± 0.0008 * [32]
Disodium creatinephosphate	0.1 [49]

* Solubility values determined in phosphate-buffered saline.

**Table 2:** Reported quantitative dissolution kinetics of creatine forms. All data are expressed in mg·cm^−2^·min^−1^ for comparability.

Creatine Form	Intrinsic Dissolution Rate Constant, mg·cm^−2^·min^−1^	Experimental Conditions
Creatine	6.92	Rotating disc method, deionized water, 37 °C [87]
Creatine monohydrate	5.18	
Di-creatine citrate	7.61	
Buffered creatine	1.19 ± 0.01	Intrinsic Dissolution Rate test, pH 2.5 buffer, room temperature [88]
Creatine monohydrate	0.99 ± 0.03	
Creatine nitrate	4.88 ± 0.06	
Buffered creatine	1.97 ± 0.02	Intrinsic Dissolution Rate test, pH 2.5 buffer, 37 °C [88]
Creatine monohydrate	1.63 ± 0.01	
Creatine nitrate	5.46 ± 0.04	
Buffered creatine	0.84 ± 0.02	Intrinsic Dissolution Rate test, pH 7.4 buffer, room temperature [88]
Creatine monohydrate	0.87 ± 0.00	
Creatine nitrate	4.65 ± 0.24	
Buffered creatine	1.62 ± 0.15	Intrinsic Dissolution Rate test, pH 7.4 buffer, 37 °C [88]
Creatine monohydrate	1.43 ± 0.08	
Creatine nitrate	5.33 ± 0.24	

## Data Availability

No new data were created or analyzed in this study. Data sharing is not applicable to this article.

## Abbreviations

The following abbreviations are used in this manuscript:

a-CCA	Co-amorphous form of creatine with citric acid
ATP	Adenosine triphosphate
BBB	Blood–brain barrier
BCAA	Branched-chain amino acids
CAGR	Compound Annual Growth Rate
c-CCA	Co-crystalline form of creatine with citric acid
CIPO	Canadian Intellectual Property Office
CM	Creatine monohydrate
CPC	Cooperative Patent Classification
DFT	Density Functional Theory
HPMC	Hydroxypropyl methylcellulose
IPC	International Patent Classification
IR	Infrared
PEG	Polyethylene glycol
PVP	Polyvinylpyrrolidone
RESS	Rapid Expansion of Supercritical Solutions
SCF	Supercritical fluids
TGA	Thermogravimetric analysis

## Appendix A

**Table A1:** Key patent developments of modified (fast-dissolving) creatine forms over the past 25 years.

№	Number	Title	Technology	Method
1	US10881630B2	Creatine oral supplementation using creatine hydrochloride salt	Chemical modification	Creatine hydrochloride synthesis
2	US8624053B2	Method to produce a stable dry ionic-bonded creatine alpha ketoglutarate of high oral absorbability		Creatine α-ketoglutarate synthesis
3	US2013096193A1 (Abandoned)	Creatine beta-alaninate: a novel salt for increasing athletic performance		Creatine β-alaninate synthesis
4	US2025019344A1 (Pending)	Creatine carbonate and methods of production and uses		Creatine carbonate synthesis
5	CN119039183A (Pending)	Creatine carbonate, preparation method thereof and creatine supplement		
6	US8466198B2	Compositions comprising creatine salts and methods of use thereof		Creatine bicarbonate synthesis
7	US7482474B2	Creatine pyroglutamic acid salts and methods for their production and use in individuals		Creatine pyroglutamate synthesis
8	US7511173B2	Creatine salt with enhanced nutritional and therapeutic efficacy and compositions containing same		Creatine glycinate synthesis
9	US2008254198A1 (Abandoned)	Method of preparing creatine ester salts and uses thereof		Organic salts of creatine ethyl ester production
10	US8546369B2	Salts of creatine imino sugar amides		Production of salts of creatine amides with iminosugars
11	US2024075002A1 (Pending)	Creatine composition and methods of using same		Creatine double salts production
12	US2007281910A1 (Abandoned)	Salicyl alcohol creatine phosphate prodrugs, compositions and uses thereof		Salicylate esters of creatine phosphate synthesis
13	US7511164B2	Creatine-fatty acids		Creatine compounds with fatty acids production
14	US9642825B2	Bio-available n-acetyl creatine species and compositions thereof		N-acetyl creatine and its derivatives synthesis
15	US10531680B2	Creatine ester pronutrient compounds and formulations		Creatine esters with alcohols synthesis
16	US8735623B2	Process for preparing creatine amides		Creatine amides synthesis
17	US8426395B2	Preparations containing creatine and imino sugars		Creatine imino sugar amides synthesis
18	US9114150B2	Stable aqueous compositions comprising bioactive creatine species		Creatinol-O-phosphate salts synthesis
19	CN101274943B	Synthetic method for disodium creatine phosphate		Phosphocreatine disodium synthesis
20	US2009098221A1 (Abandoned)	Creatine ascorbyl derivatives and methods of use thereof		Creatine ascorbyl phosphate and its derivatives synthesis
21	AU2018210739B2	Phenylcreatine, its use and method for its production		Phenyl creatine synthesis
22	US11970435B2	Taste-modified creatine salts, compounds, compositions and uses thereof		Production of creatine salts with sweeteners
23	US7772428B2	Creatine hydroxycitric acids salts and methods for their production and use in individuals		Creatine hydroxycitrate synthesis
24	USRE43029E	Process for preparing a creatine heterocyclic acid salt and method of use		Creatine orotate and its derivatives production
25	US7301051B2	Creatine salts and method of making same		Synthesis of creatine salts with dicarboxylic acids
26	US11753369B2	Creatine prodrugs, compositions and methods of use thereof		Synthesis of salts of intramolecular esterified creatine amides + microcapsules/liposomes
27	JP2007039408A (Withdrawn)	Pulverized creatine and method for producing the same	Micronization	Wet milling in bead mills with non-aqueous solvents
28	US2002151593A1 (Abandoned)	Water-soluble creatine monohydrate formulations and process for their preparation		Micronization with dextrose, flavors, and colorants
29	JP3892610B2	Production of food or medicine comprising granular form and tablet form		Micronization + granulation
30	DE102022114966A1 (Pending)	Water-soluble creatine agglomerate	Granulation	Granulation with maltodextrin
31	US2007071815A1 (Abandoned)	Oral formulation of creatine derivatives and method of manufacturing same		Granulation of creatine ethyl ester with excipients
32	US12280067B2	Formulations of creatine and cyclodextrin exhibiting improved bioavailability		Granulation with subsequent coating of granules
33	RU2752141C1	Method for producing granulated sugar-containing product for sports nutrition		Granulation with sugar and leucine
34	CN104432095A (Pending)	Creatine instant powder and preparation method thereof	Amorphization	Amorphization with sucrose/glucose
35	US12208076B2	Mechanosynthesis of a co-amorphous formulation of creatine with citric acid and humidity-mediated transformation into a co-crystal		Amorphization and co-crystallization (parallel)
36	EP2926669B1	Creatine-protein matrix and method for producing said matrix		Amorphization combined with modified solid dispersion
37	US10231933B2	Enteric coated, soluble creatine and polyethylene glycol composition for enhanced skeletal uptake of oral creatine	Solid Dispersions	Solid dispersion with PEG
38	US6689299B2	Process for producing solid creatine dosage forms and dosage forms obtainable thereby		Solid dispersion with PVP
39	US9445622B2	Compositions and methods for improving creatine solubility and stability		Modified solid dispersion
40	RU2596485C1	Method of producing creatine nanocapsules in gellan gum	Capsules	Nanocapsules
41	RU2538695C1	Method of encapsulating creatine having supramolecular properties		Microcapsules
42	CN101732263A (Pending)	Creatine phosphate sodium freeze-dried preparation and method for preparing same	Other Technological Approaches	Lyophilization with fillers
43	CN102872061A (Pending)	Composition of creatine phosphate and arginine		Solubilization by arginine

## References

[1] Grand View Research (2023). Sports Nutrition Market Size, Share & Trends Analysis Report by Product Type (Sports Supplements, Sports Drinks), by Formulation, by Consumer Group, by Sales Channel, by Region, and Segment Forecasts, 2024–2030.

[2] Jäger R., Kerksick C.M., Campbell B.I., Cribb P.J., Wells S.D., Skwiat T.M., Purpura M., Ziegenfuss T.N., Ferrando A.A., Arent S.M. (2017). International Society of Sports Nutrition Position Stand: Protein and Exercise. J. Int. Soc. Sports Nutr..

[3] Knapik J.J., Steelman R.A., Hoedebecke S.S., Austin K.G., Farina E.K., Lieberman H.R. (2016). Prevalence of Dietary Supplement Use by Athletes: Systematic Review and Meta-Analysis. Sports Med..

[4] Wang Z., Qiu B., Li R., Han Y., Petersen C., Liu S., Zhang Y., Liu C., Candow D.G., Del Coso J. (2024). Effects of Creatine Supplementation and Resistance Training on Muscle Strength Gains in Adults <50 Years of Age: A Systematic Review and Meta-Analysis. Nutrients.

[5] Desai I., Wewege M.A., Jones M.D., Clifford B.K., Pandit A., Kaakoush N.O., Simar D., Hagstrom A.D. (2024). The Effect of Creatine Supplementation on Resistance Training-Based Changes to Body Composition: A Systematic Review and Meta-Analysis. J. Strength Cond. Res..

[6] Kreider R.B., Kalman D.S., Antonio J., Ziegenfuss T.N., Wildman R., Collins R., Candow D.G., Kleiner S.M., Almada A.L., Lopez H.L. (2017). International Society of Sports Nutrition Position Stand: Safety and Efficacy of Creatine Supplementation in Exercise, Sport, and Medicine. J. Int. Soc. Sports Nutr..

[7] Gualano B., Rawson E.S., Candow D.G., Chilibeck P.D. (2016). Creatine Supplementation in the Aging Population: Effects on Skeletal Muscle, Bone and Brain. Amino Acids.

[8] Dolan E., Gualano B., Rawson E.S. (2019). Beyond Muscle: The Effects of Creatine Supplementation on Brain Creatine, Cognitive Processing, and Traumatic Brain Injury. Eur. J. Sport. Sci..

[9] Chilibeck P.D., Kaviani M., Candow D.G., Zello G.A. (2017). Effect of Creatine Supplementation During Resistance Training on Lean Tissue Mass and Muscular Strength in Older Adults: A Meta-Analysis. Open Access J. Sports Med..

[10] Robert M.D. (2023). Creatine Nutritional Supplement. U.S. Patent.

[11] Kreider R.B., Jäger R., Purpura M. (2022). Bioavailability, Efficacy, Safety, and Regulatory Status of Creatine and Related Compounds: A Critical Review. Nutrients.

[12] Candow D.G., Forbes S.C., Roberts M.D., Roy B.D., Antonio J., Smith-Ryan A.E., Rawson E.S., Gualano B., Roschel H. (2022). Creatine O’Clock: Does Timing of Ingestion Really Influence Muscle Mass and Performance?. Front. Sports Act. Living.

[13] Jäger R., Purpura M., Shao A., Inoue T., Kreider R.B. (2011). Analysis of the Efficacy, Safety, and Regulatory Status of Novel Forms of Creatine. Amino Acids.

[14] Antonio J., Ciccone V. (2013). The Effects of Pre versus Post Workout Supplementation of Creatine Monohydrate on Body Composition and Strength. J. Int. Soc. Sports Nutr..

[15] Persky A.M., Brazeau G.A. (2001). Clinical Pharmacology of the Dietary Supplement Creatine Monohydrate. Pharmacol. Rev..

[16] Blagden N., de Matas M., Gavan P.T., York P. (2007). Crystal Engineering of Active Pharmaceutical Ingredients to Improve Solubility and Dissolution Rates. Adv. Drug Deliv. Rev..

[17] Williams H.D., Trevaskis N.L., Charman S.A., Shanker R.M., Charman W.N., Pouton C.W., Porter C.J.H. (2013). Strategies to Address Low Drug Solubility in Discovery and Development. Pharmacol. Rev..

[18] Kawakami K. (2012). Modification of Physicochemical Characteristics of Active Pharmaceutical Ingredients and Application of Super-Saturatable Dosage Forms for Improving Bioavailability of Poorly Absorbed Drugs. Adv. Drug Deliv. Rev..

[19] Serajuddin A.T.M. (2007). Salt Formation to Improve Drug Solubility. Adv. Drug Deliv. Rev..

[20] Miller D.W., Vennerstrom J.L., Faulkner M.C., Vireo Systems, Inc., Board of Regents of the University of Nebraska (2021). Creatine Oral Supplementation Using Creatine Hydrochloride Salt. U.S. Patent.

[21] Silber M., Yusufov T. (2014). Method to Produce a Stable Dry Ionic-Bonded Creatine Alpha Ketoglutarate of High Oral Absorbability. U.S. Patent.

[22] Kneller B.W. (2013). Creatine Beta-Alaninate: A Novel Salt for Increasing Athletic Performance. U.S. Patent.

[23] ThermoLife International LLC (2025). Creatine Carbonate and Methods of Production and Uses. U.S. Patent.

[24] Waterstone Pharmaceuticals Wuhan Co., Ltd., Hubei Waterstone Bio Pharmaceutical Tech Co., Ltd., Hubei Shihe Medicine Tech Co., Ltd (2024). Creatine Carbonate, Preparation Method Thereof and Creatine Supplement. Chinese Patent.

[25] Kneller B. (2013). Compositions Comprising Creatine Salts and Methods of Use Thereof. U.S. Patent.

[26] New Cell Formulations, Ltd (2009). Creatine Pyroglutamic Acid Salts and Methods for Their Production and Use in Individuals. U.S. Patent.

[27] Iovate T & P Inc (2009). Creatine Salt with Enhanced Nutritional and Therapeutic Efficacy and Compositions Containing Same. U.S. Patent.

[28] Bio-Engineered Supplements & Nutrition Inc (2008). Method of Preparing Creatine Ester Salts and Uses Thereof. U.S. Patent.

[29] Heuer M., Molino M., MacDougall J., Northern Innovations Holding Corp (2013). Salts of Creatine Imino Sugar Amides. U.S. Patent.

[30] NanoChem Solutions Inc (2024). Creatine Composition and Methods of Using Same. U.S. Patent.

[31] Xenoport, Inc (2007). Salicyl Alcohol Creatine Phosphate Pro-Drugs, Compositions and Uses Thereof. U.S. Patent.

[32] Board of Regents of the University of Nebraska (2020). Creatine Ester Pronutrient Compounds and Formulations. U.S. Patent.

[33] Multi Formulations Ltd (2009). Creatine-Fatty Acids. U.S. Patent.

[34] 4141 Holdings, LLC (2017). Bio-Available N-acetyl Creatine Species and Compositions Thereof. U.S. Patent.

[35] Burov S.V., Veselkina O.S., Leko M.V. (2014). Process for Preparing Creatine Amides. U.S. Patent.

[36] Heuer M.A., Molino M., MacDougall J., Northern Innovations Holding Corp (2013). Preparations Containing Creatine and Imino Sugars. U.S. Patent.

[37] Owoc J.H., Jho Intellectual Property Holdings LLC (2015). Stable Aqueous Compositions Comprising Bioactive Creatine Species. U.S. Patent.

[38] Shanghai Cirui Medicine Tech Co., Ltd (2011). Synthetic Method for Disodium Creatine Phosphate. Chinese Patent.

[39] Nivaggioli B.T. (2009). Creatine Ascorbyl Derivatives and Methods of Use Thereof. U.S. Patent.

[40] Dukhovlinov I.V., Alekseev A.V. (2021). Phenylcreatine, Its Use and Method for Its Production. Australian Patent.

[41] Augusta University Research Institute Inc (2024). Taste-Modified Creatine Salts, Compounds, Compositions and Uses Thereof. U.S. Patent.

[42] Northern Innovations Holding Corp (2010). Creatine Hydroxycitric Acids Salts and Methods for Their Production and Use in Individuals. U.S. Patent.

[43] ABio Engineered Supplements & Nutrition Inc (2011). Process for Preparing a Creatine Heterocyclic Acid Salt and Method of Use. U.S. Patent.

[44] Starmark Lab (2007). Creatine Salts and Method of Making Same. U.S. Patent.

[45] Ultragenyx Pharmaceutical Inc (2023). Creatine Prodrugs, Compositions and Methods of Use Thereof. U.S. Patent.

[46] Gufford B.T., Sriraghavan K., Miller N.J., Miller D.W., Gu X., Vennerstrom J.L., Robinson D.H. (2010). Physicochemical Characterization of Creatine N-methylguanidinium Salts. J. Diet. Suppl..

[47] Flamma SPA (1998). Hydrosoluble Organic Salts of Creatine. European Patent.

[48] Llewellyn W.C. (2004). Dicreatine Maleate and Method for Making Same. U.S. Patent.

[49] Chemical Book (2009). Creatine Phosphate Disodium Salt. https://www.chemicalbook.com/ChemicalProductProperty_EN_CB9668056.htm.

[50] Rasenack N., Müller B.W. (2004). Micron-Size Drug Particles: Common and Novel Micronization Techniques. Pharm. Dev. Technol..

[51] Unitec Foods Co., Ltd (2007). Pulverized Creatine and Method for Producing the Same. Japanese Patent.

[52] Stitley J., Faranetta A. (2002). Water-Soluble Creatine Monohydrate Formulations and Process for Their Preparation. U.S. Patent.

[53] Ezaki Glico Co., Ltd (2007). Production of Food or Medicine Comprising Granular Form and Tablet Form. Japanese Patent.

[54] Hezave A., Aftab S., Esmaeilzadeh F. (2010). Micronization of Creatine Monohydrate via Rapid Expansion of Supercritical Solution (RESS). J. Supercrit. Fluids.

[55] Shanmugam S. (2015). Granulation Techniques and Technologies: Recent Progresses. Bioimpacts.

[56] AlzChem Trostberg GmbH (2023). Water-Soluble Creatine Agglomerate. German Patent.

[57] Byrd E.A. (2007). Oral Formulation of Creatine Derivatives and Method of Manufacturing Same. U.S. Patent.

[58] Phenolics LLC (2025). Formulations of Creatine and Cyclodextrin Exhibiting Improved Bioavailability. U.S. Patent.

[59] Federalnoe Gosudarstvennoe Byudzhetnoe Obrazovatelnoe Uchrezhdenie Vysshego Obrazovaniya “Moskovskij Gosudarstvennyj Universitet Tekhnologij i Upravleniya Imeni K.G. Razumovskogo (Pervyj Kazachij Universitet)” (2021). Method for Producing Granulated Sugar-Containing Product for Sports Nutrition. Russian Patent.

[60] Thapa P., Choi D.H., Kim M.S., Jeong S.H. (2019). Effects of Granulation Process Variables on the Physical Properties of Dosage Forms by Combination of Experimental Design and Principal Component Analysis. Asian J. Pharm. Sci..

[61] Vandevivere L., Vangampelaere M., Portier C., de Backere C., Häusler O., De Beer T., Vervaet C., Vanhoorne V. (2021). Identifying Critical Binder Attributes to Facilitate Binder Selection for Efficient Formulation Development in a Continuous Twin Screw Wet Granulation Process. Pharmaceutics.

[62] Tambe S., Jain D., Meruva S.K., Rongala G., Juluri A., Nihalani G., Mamidi H.K., Nukala P.K., Bolla P.K. (2022). Recent Advances in Amorphous Solid Dispersions: Preformulation, Formulation Strategies, Technological Advancements and Characterization. Pharmaceutics.

[63] Guner G., Amjad A., Berrios M., Kannan M., Bilgili E. (2023). Nanoseeded Desupersaturation and Dissolution Tests for Elucidating Supersaturation Maintenance in Amorphous Solid Dispersions. Pharmaceutics.

[64] Wlodarski K., Sawicki W., Paluch K., Tajber L., Grembecka M., Hawelek L., Wojnarowska Z., Grzybowska K., Talik E., Paluch M. (2014). The Influence of Amorphization Methods on the Apparent Solubility and Dissolution Rate of Tadalafil. Eur. J. Pharm. Sci..

[65] Baghel S., Cathcart H., O’Reilly N.J. (2016). Polymeric Amorphous Solid Dispersions: A Review of Amorphization, Crystallization, Stabilization, Solid-State Characterization, and Aqueous Solubilization of Biopharmaceutical Classification System Class II Drugs. J. Pharm. Sci..

[66] Kim D., Kim Y., Tin Y.-Y., Soe M.-T.P., Ko B., Park S., Lee J. (2021). Recent Technologies for Amorphization of Poorly Water-Soluble Drugs. Pharmaceutics.

[67] Univ Jiangnan (2015). Creatine Instant Powder and Preparation Method Thereof. Chinese Patent.

[68] Southern Methodist University (2025). Mechanosynthesis of a Co-Amorphous Formulation of Creatine with Citric Acid and Humidity-Mediated Transformation into a Co-Crystal. U.S. Patent.

[69] Alzchem A.G. (2017). Creatine-Protein Matrix and Method for Producing Said Matrix. European Patent.

[70] Joshi M., Tiwari G., Tiwari R., Pandey S.R., Pandey P., Rai A.K. (2012). Solid Dispersion: A Technology for the Improvement of Oral Bioavailability of Poorly Soluble Drugs. Curr. Pharma Res..

[71] Cryxa LLC (2019). Enteric Coated, Soluble Creatine and Polyethylene Glycol Composition for Enhanced Skeletal Uptake of Oral Creatine. U.S. Patent.

[72] BASF Aktiengesellschaft (2004). Process for Producing Solid Creatine Dosage Forms and Dosage Forms Obtainable Thereby. U.S. Patent.

[73] Glanbia Nutritionals (Ireland) Ltd (2016). Compositions and Methods for Improving Creatine Solubility and Stability. U.S. Patent.

[74] Altemimi A.B., Farag H.A.M., Salih T.H., Awlqadr F.H., Al-Manhel A.J.A., Vieira I.R.S., Conte-Junior C.A. (2024). Application of Nanoparticles in Human Nutrition: A Review. Nutrients.

[75] Kothamasu P., Kanumur H., Ravur N., Maddu C., Parasuramrajam R., Thangavel S. (2012). Nanocapsules: The Weapons for Novel Drug Delivery Systems. Bioimpacts.

[76] Peanparkdee M., Iwamoto S., Yamauchi R. (2016). Microencapsulation: A Review of Applications in the Food and Pharmaceutical Industries. Rev. Agric. Sci..

[77] Yusuf A., Almotairy A.R.Z., Henidi H., Alshehri O.Y., Aldughaim M.S. (2023). Nanoparticles as Drug Delivery Systems: A Review of the Implication of Nanoparticles’ Physicochemical Properties on Responses in Biological Systems. Polymers.

[78] Yang X.C., Samanta B., Agasti S.S., Jeong Y., Zhu Z., Rana S., Miranda O.R., Rotello V.M. (2011). Drug Delivery Using Nanoparticle-Stabilized Nanocapsules. Angew. Chem. Int. Ed. Engl..

[79] Krolevets A.A. (2016). Method of Producing Creatine Nanocapsules in Gellan Gum. Russian Patent.

[80] Krolevets A.A., Bogachev I.A., Nikitin K.S., Boyko E. (2015). Method of Encapsulating Creatine Having Supramolecular Properties. Russian Patent.

[81] Trotier-Faurion A., Passirani C., Béjaud J., Dézard S., Valayannopoulos V., Taran F., de Lonlay P., Benoit J.-P., Mabondzo A. (2015). Dodecyl Creatine Ester and Lipid Nanocapsule: A Double Strategy for the Treatment of Creatine Transporter Deficiency. Nanomedicine.

[82] Bordim K.A.Q., Carvalho Júnior J.A.B., Ramos V.S., Tavares M.I.B. (2025). Nanoencapsulation of Creatine: Unlocking Hydrophilic Bioactive Potential Through Double Emulsification by Solvent Diffusion. J. Appl. Polym. Sci..

[83] Yang J. (2010). Creatine Phosphate Sodium Freeze-Dried Preparation and Method for Preparing Same. Chinese Patent.

[84] Wang L. (2013). Composition of Creatine Phosphate and Arginine. Chinese Patent.

[85] Giese M.W., Lecher C.S. (2009). Qualitative In Vitro NMR Analysis of Creatine Ethyl Ester Pronutrient in Human Plasma. Int. J. Sports Med..

[86] Gufford B.T., Ezell E.L., Robinson D.H., Miller D.W., Miller N.J., Gu X., Vennerstrom J.L. (2013). pH-Dependent Stability of Creatine Ethyl Ester: Relevance to Oral Absorption. J. Diet. Suppl..

[87] Ganguly S., Jayappa S., Dash A.K. (2003). Evaluation of the Stability of Creatine in Solution Prepared from Effervescent Creatine Formulations. AAPS PharmSciTech.

[88] ThermoLife Int LLC (2021). Amino Acid Compositions. U.S. Patent.

